# Improved Macro- and Micronutrient Supply for Favorable Growth and Metabolomic Profile with Standardized Parenteral Nutrition Solutions for Very Preterm Infants

**DOI:** 10.3390/nu14193912

**Published:** 2022-09-21

**Authors:** Alida Kindt, Yvonne Kraus, Livia Dahlia Rasp, Kai M. Foerster, Narges Ahmidi, Andreas W. Flemmer, Susanne Herber-Jonat, Florian Heinen, Heike Weigand, Thomas Hankemeier, Berthold Koletzko, Jan Krumsiek, Juergen Babl, Anne Hilgendorff

**Affiliations:** 1 Institute of Computational Biology, Helmholtz Zentrum München, 85764 Oberschleißheim, Germany; 2 Metabolomics and Analytics Center, Leiden Academic Centre for Drug Research, Leiden University, 2333 AL Leiden, The Netherlands; 3 Center for Comprehensive Developmental Care (CDeCLMU), Division of Pediatric Neurology, Developmental Medicine and Social Pediatrics, Department of Pediatrics, Dr von Hauner Children's Hospital, Munich University Hospital, Ludwig Maximilians University, 80336 Munich, Germany; 4 Comprehensive Pneumology Center, Helmholtz Zentrum München, Member of the German Lung Research Center (DZL), 81377 Munich, Germany; 5 Department of Neonatology, Perinatal Center, Dr. von Hauner Children’s Hospital, Ludwig-Maximilians University, 80337 Munich, Germany; 6 Division of Metabolic and Nutritional Medicine, Department of Paediatrics, Dr. von Hauner Children’s Hospital University Hospital, Ludwig-Maximilians University, 81377 Munich, Germany; 7 Institute for Computational Biomedicine, Englander Institute for Precision Medicine, Department of Physiology and Biophysics, Weill Cornell Medicine, New York, NY 10065, USA; 8 Pharmacy of the University Hospital, Ludwig-Maximilians University, 81377 Munich, Germany

**Keywords:** preterm, parenteral nutrition, growth, macronutrients, micronutrients, electrolytes

## Abstract

Very preterm infants are at high risk for suboptimal nutrition in the first weeks of life leading to insufficient weight gain and complications arising from metabolic imbalances such as insufficient bone mineral accretion. We investigated the use of a novel set of standardized parenteral nutrition (PN; *MUC PREPARE*) solutions regarding improving nutritional intake, accelerating termination of parenteral feeding, and positively affecting growth in comparison to individually prescribed and compounded PN solutions. We studied the effect of *MUC PREPARE* on macro- and micronutrient intake, metabolism, and growth in 58 very preterm infants and compared results to a historic reference group of 58 very preterm infants matched for clinical characteristics. Infants receiving *MUC PREPARE* demonstrated improved macro- and micronutrient intake resulting in balanced electrolyte levels and stable metabolomic profiles. Subsequently, improved energy supply was associated with up to 1.5 weeks earlier termination of parenteral feeding, while simultaneously reaching up to 1.9 times higher weight gain at day 28 in extremely immature infants (<27 GA weeks) as well as overall improved growth at 2 years of age for all infants. The use of the new standardized PN solution MUC PREPARE improved nutritional supply and short- and long-term growth and reduced PN duration in very preterm infants and is considered a superior therapeutic strategy.

## 1. Introduction

About 1% of infants are born very preterm, i.e., before 32 week gestational age (GA) [1]. Despite significant advances in perinatal medicine that led to survival rates of up to 70% in the most immature infants [2], management of nutrition and postnatal growth remains a challenge in this high-risk patient population even for experienced perinatal centers.

Adequate nutritional support strategies for preterm infants are important for meeting their macro- and micronutrient needs for health growth and development and are related to the time to regain birth weight and prevalence of postnatal complications including the rate of necrotizing enterocolitis (NEC), nosocomial sepsis, and bronchopulmonary dysplasia (BPD) [3,4,5]. Importantly, substrate—in particular, higher protein—and energy intake during the first 7 days of life is associated with improved neurodevelopmental outcomes at 18 months of age [6,7]. In line with this, weight gain velocity during neonatal intensive care is associated with a significant positive effect on neurodevelopmental outcome and growth in the first years of life [8], with adequate nutrition and somatic growth revealing favorable effects on brain growth and white matter maturation [9]. Furthermore, early and aggressive introduction of enteral feeding was associated with improved growth developmental outcomes [6], even though the transition period of parenteral to enteral feeding is precarious and nutrient deficits can occur [10].

As a result, optimization of nutritional intake while compensating for immaturity-associated complications has become one of the most important goals in neonatal care [4,11,12,13,14,15,16,17,18,19] which requires the use of parenteral nutrition (PN) from day 1 of life in very preterm infants until sufficient enteral nutrition can be established [5]. Given the vulnerability of very preterm infants, the highest hygiene standards for both production and application of PN solutions must be met in this high-risk population [15].

Recommendations for PN in the very preterm infant changed significantly over the past years, requiring perinatal centers to adapt their PN regimen [20,21]. Next to training programs addressing composition and application of PN, standardized solutions emerged to meet nutritional needs and hygiene standards more broadly in the preterm population by reducing both delays and errors or inconsistencies when prescribing PN [22,23]. Standardized PN solutions for infants marketed by commercial companies, however, are not specifically designed to meet the needs of very preterm infants but are mostly designed to approximately meet the needs of a broader group of infants.

We therefore developed and evaluated standardized PN solutions (MUniCh PREterm PAREnteral solution (*MUC PREPARE*)), meeting the specific nutritional needs of very preterm infants in line with established recommendations [18,22,24]. We assessed the effect of our newly developed PN solutions on macro- and micronutrient intake, plasma electrolyte levels and metabolites, as well as somatic growth until discharge and at two years of age in very preterm infants in comparison to a historic reference group with matched pairs that had received individually designed PN solutions.

## 2. Materials and Methods

### 2.1. Study Population

The cohort comprises 116 very preterm infants ≤ 32 weeks GA born at the Perinatal Center of the University Hospital of Ludwig-Maximilians-Universität (LMU), Campus Grosshadern. Exclusion criteria were severe congenital malformations, chromosomal abnormalities, and metabolic diseases. Written informed parental consent was obtained for all study infants (AIRR study cohort: Munich #195-07, Munich # 492-15). The study was registered at the German Registry for Clinical Studies (DRKS00004600). Of the 116 infants, 58 preterm infants born between 24 January 2012 and 30 April 2014 were prospectively enrolled and received the standardized PN regimen (study cohort: median birth weight 989.3 g (interquartile range [IQR]: 722.5–1275 g)). A retrospectively recruited second cohort of 58 infants born between 17 January 2007 and 5 December 2009 was established based on pairs matched for gestational age, birth weight, birth weight percentile, gender, multiples, presence of infection at birth, grade of respiratory distress syndrome, intraventricular hemorrhage, and persistent ductus arteriosus (reference cohort: median birth weight 990.9 g (IQR: 745–1222.5 g)). Table 1 presents the demographic characteristics of the two patient cohorts and the respective subgroups divided by age. The two cohorts were similar for clinical characteristics and complications except for a higher BPD incidence in the study cohort (Fisher’s exact test *p*-value = 1.04 × 10^−7^).

### 2.2. Development of the Standardized Parenteral Nutrition Solution MUC PREPARE

The standardized PN solution (Munich Preterm Parenteral feeding solution (*MUC PREPARE*)) was designed in cooperation with the Pharmacy of the University Hospital to fulfil the ESPGHAN (European Society for Paediatric Gastroenterology Hepatology and Nutrition) and ESPEN (European Society for Clinical Nutrition and Metabolic Care) guidelines [24] while achieving long-lived chemical and physical stability of its contents. The PN solutions are tailored to (i) meet the different stages of intake and fluid balance after birth [13,24] by the means of three different ranges of macro- and micronutrient concentrations and (ii) enable both peripheral and central venous application. Composition of the PN solutions furthermore addressed the rapidly changing nutritional needs including adaptations necessary due to expected complications in the first weeks of life. These prerequisites resulted in a total of nine standardized PN solutions *MUC PREPARE* (Table 2 and Table 3) together with three solutions containing commercial fat emulsions and vitamins (Table 4 and Table 5). Solution 1 is designed for the first day of life allowing for a reduced supply of macro- and micronutrients and overall fluid intake as recommended. Solution 2 is designed for increased parenteral supply of macro- and micronutrients in infants exhibiting significant intra- and interindividual variability in the parenteral supply needed due to fluid and metabolic imbalance and clinically critical conditions. Solution 3 is designed to meet stabilized conditions that allow for the transition to enteral feeding. In order to allow for optimal protein supply in infants with slightly restricted carbohydrate supply due to hyperglycemia, the solutions for central venous application were divided into “G(+)” and “G(−)” referring to different glucose concentrations. Each infusion is composed of one main infusion bag that can be combined with different bypass solutions for additional amino acid, glucose, or water supply. Trace element solutions are delivered in a syringe and added to the main infusion bag right before application. The standardized PN solutions furthermore fulfil the requirements of the updated ESPGHAN guidelines [18,22], that recommend a lower calorie intake (45–55 kcal/kg bw/day) in the first days of life, followed by an increase to 90–120 kcal/kg bw/day in the intermediate phase based on a presumed lower energy consumption due to low amounts of enteral feeding.

### 2.3. Parenteral Nutrition

Application of PN for both study groups followed the ESPGHAN guidelines for pediatric PN published in 2005 supported by a structured recommendation plan displayed at the desk of the prescribing physician. All prescriptions were carried out with the software ‘Visite2000′ [25]. Every prescribing physician received a 2-week training prior to the first prescription, followed by update training twice a year and continuous supervision through a senior attending neonatologist. PN was prescribed every morning and prepared by the Pharmacy of the University Hospital according to Annex 1, EUGMP (Good Manufacturing Practice) guidelines. All PN nutrition regimens contained the solutions outlined above including fat emulsion (Table 2 and Table 3) until enteral feeding contained the macronutrient intake recommended. While the study cohort (*n =* 58) received standardized PN solutions as outlined above (Table 2, Table 3, Table 4 and Table 5), the reference cohort (*n =* 58) received individually designed and compounded PN solutions. Both standardized and individual PN solutions were adapted with respect to volume and electrolyte content as clinically indicated.

### 2.4. Enteral Feeding

Enteral feeding was initiated on the first day of life along with PN in all study infants and based on fortified own mothers’ milk or donor milk (fortifier FM 85 (Nestlé, Frankfurt M, Germany) or, in case human milk was not available, preterm formula (study cohort: Aptamil Prematil [Milupa, Friedrichsdorf, Germany], reference cohort: Beba FG [Nestlé, Frankfurt M, Germany])). Enteral feeding was adjusted according to ESPGHAN recommendations as available at the time (study cohort [26], reference cohort [27], Supplementary Table S1, overview of differences between cohorts, Supplementary Table S2, Summary table of provided nutrients). Iron supplementation was initiated when enteral feeding exceeded 75% of recommended energy intake.

### 2.5. Clinical Monitoring

Data collection was performed at 8 timepoints (days of life 1, 3, 7, 14, 21, and 28 and respective age of 32- and 36-weeks GA) and comprised the following variables: intake of total fluids, calories, carbohydrates, proteins, lipids, and electrolytes via parenteral (and enteral) feeding. Nutritional supply composition was expressed with respect to the ratio enteral/parenteral (% of total mL). Serum parameters monitored included glucose, sodium, potassium, lactate, calcium, phosphate, and magnesium, all of which were determined by blood gas analysis (ABL-Radiometer, Copenhagen, Denmark) every day to twice a week. Organ function and infections were assessed by weekly analysis of differential blood count, C-reactive protein, liver parameters, and creatinine. Blood triglycerides were measured once or twice a month depending on routine blood work and signs of metabolic imbalance. In addition, acute and chronic complications were monitored: early onset and late onset neonatal infection, respiratory distress syndrome (RDS), intraventricular hemorrhage (IVH), and persistent ductus arteriosus (PDA) (Table 1). Growth parameters were assessed by the attending intensive care nurse using standardized procedures and calibrated scales; relative growth was calculated using standardized growth percentile charts, i.e., weight-for-age z-score charts [28]. Weight gain [g], growth of head circumference (HC (cm)) and body length (BL (cm)) were calculated as a percentage of the observed parameters at day 28 to birth. Somatic growth, i.e., weight gain, growth of head circumference, and body length was reassessed at 24 months and corrected for degree of prematurity.

### 2.6. Metabolomics

Levels of 397 metabolites (amino acids, biogenic amines, lipids, and acylcarnitines) in blood plasma samples of 22 infants (study cohort: median: 2.5 days of life, range: 0–30) were measured by ultra-high-performance LC tandem mass spectrometry (UPLC-MS/MS; BioMedical Metabolomics Facility Leiden at the Leiden University). Relative abundances of the metabolites were reported and log_2_ transformed prior to statistical analysis. After exclusion of metabolites (*n =* 138) and/or samples (*n =* 3) with >20% missingness, a total of 259 metabolites and 19 samples were analyzed. Remaining missing values were imputed using kNN-based imputation [29], and one outlier detected in the PCA analysis was removed. Association of metabolite levels with changes in nutritional intake (macronutrients including total fluid and total calorie intake, percentage of carbohydrate, fat and protein to overall calorie consumption, concentration of added micronutrients (natrium, potassium, calcium, magnesium, chloride, and phosphate)) was analyzed by linear regression correcting for gender and GA. All *p*-values were corrected for multiple testing using the Benjamini–Hochberg procedure [30].

### 2.7. Statistical Analyses

Nutritional intakes, serum, and urinary excretion levels for electrolytes over the first two weeks of life were calculated for parenteral and enteral nutrition regimen per (i) individual patient, (ii) cohort, and (iii) GA subgroups (< or >27 weeks GA). Parenteral and enteral nutrition was expressed as the mean percentage of total supply at each time point. A sample was labelled as belonging to either the parenteral or enteral nutrition regimen when the respective nutritional supply exceeded 50% of the total fluid intake. Group differences were calculated using the Wilcoxon rank sum test. Feature selection of nutritional and clinical variables for growth parameters was performed using lasso regression with the primary outcome ‘growth’ (in relation to birth values). Pearson correlations were used to compare percentile values. Analysis was performed in all infants with further stratification for GA, i.e., < or >27 weeks GA. Standard infusion solutions and SOPs are detailed in the supplementary material and summarized in Table 2, Table 3, Table 4 and Table 5 (composition of the MUC PREPARE solutions).

## 3. Results

Preterm infants in the study and the reference cohort were well matched for clinical characteristics such as GA, birth weight, gender, and presence of infection. No significant difference between the two cohorts was observed for critical perinatal variables.

### 3.1. Improved Early Postnatal Energy and Nutrient Supply with the Standardized PN Solution MUC PREPARE in Very Premature Infants

**Figure 1:**
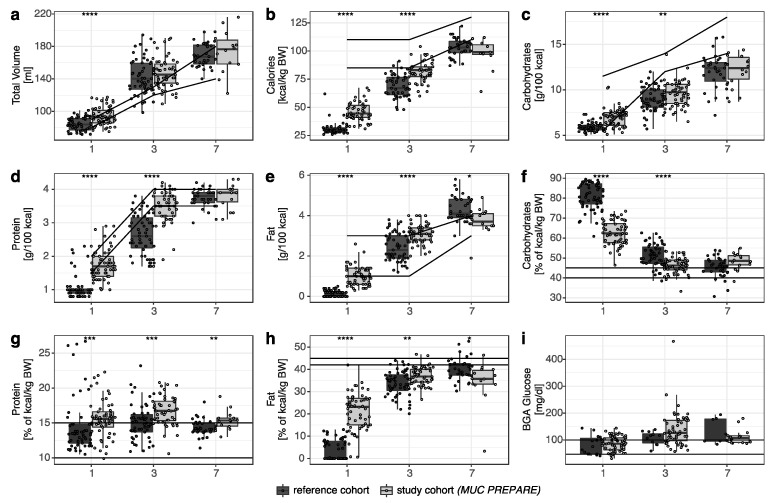
Comparison between the reference cohort (dark grey) and the study cohort (light grey; *MUC PREPARE*) for (**a**) total volume (mL), (**b**) calories (kcal/kg BW), supply shown in g/100 kcal for (**c**) carbohydrates, (**d**) protein, and (**e**) fat, (**f**–**h**) supply expressed as percentage of total calorie intake supply. (**i**) BGA glucose levels (mg/dL). Recommendations are shown as horizontal lines (ESPGHAN 2005 [24]). * *p* < 0.05, ** *p* < 0.01, *** *p* < 0.001, **** *p* < 0.0001. Study cohort (*MUC PREPARE*): 2012–2014, reference cohort: 2007–2009.

Application of the standardized PN solution *MUC PREPARE* based on the ESPGHAN guidelines resulted in overall comparable fluid intake that only differed at day 1 (10% higher in the study cohort, *p* = 1.03 × 10^−6^, Figure 1a). Infants who received the standardized PN solution *MUC PREPARE* received more than 1.5-fold higher amino acid and up to 1.5 times more energy intakes on day 1 (FC 1.5, *p* < 2.2 × 10^−16^) and day 3 of life (FC 1.2, *p* = 8.26 × 10^−10^, Figure 1b), in line with a 5-fold increase in the number of infants meeting the recommended nutrient intake in this group (*n =* 17 of 55 (study cohort, 2012–14) vs. *n =* 3 of 53 (reference cohort, 2007–09); day 3 of life). The updated ESPGHAN guidelines differing in recommended calorie intake in the first days of life are met in the study cohort received with an average intake of 46.06 ± 8.22, 81.93 ± 9.00, and 97.36 ± 13 on days 1, 3, and 7 of life, respectively.

The amino acid and higher energy intake in the first 3 days of life (Figure 1b) resulted from a balanced increase in all three macronutrients with significantly increased amino acid, lipid, and glucose intake in the *MUC PREPARE* study group until day 3 of life when compared to the reference cohort (Figure 1c–e, Supplementary Table S3).

Balanced energy supply was furthermore reflected by the significantly increased number of infants that reached the recommended relation of carbohydrate to total calorie intake (40–45% of kcal/kg BW) in the study group as early as day 3 of life (30% (study cohort) vs. 9% (reference cohort), *p*-value = 6.52 × 10^−7^, Figure 1f). The number of infants that reached the recommended protein supply was higher in infants receiving the standardized PN solution (day 1: 15.63% (study cohort) vs. 14.41% (reference cohort); day 3: 16.72% (study cohort) vs. 15.13% (reference cohort), Figure 1g). The number of infants that reached the recommended lipid supply was up to 5-fold higher when infants received the standardized PN solution at day 1 and day 3 of life (day 1: 21.3% (study cohort (*MUC PREPARE*)) vs. 4.1% (reference cohort); day 3: 36.9% (study cohort) vs. 33.7% (reference cohort), Figure 1h).

**Figure 2:**
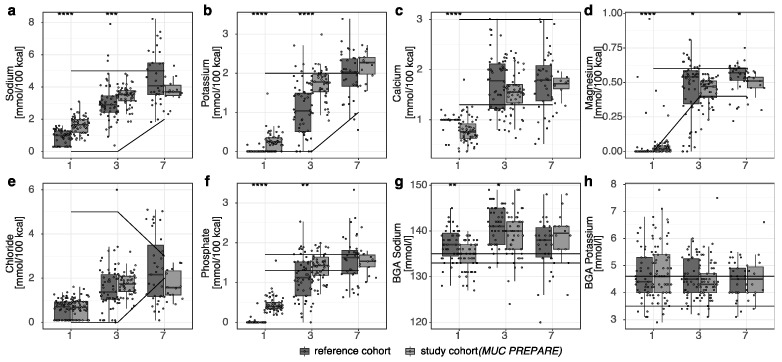
(**a**–**f**) Electrolyte supply in the parenteral diet regimens. (**g**,**h**) Serum levels of sodium and potassium. Recommendations shown as horizontal lines (ESPGHAN 2005 [24]). * *p* < 0.05, ** *p* < 0.01, *** *p* < 0.001, **** *p* < 0.0001. Study cohort (*MUC PREPARE*): 2012–2014, reference cohort: 2007–2009.

Regarding the electrolytes sodium, potassium, and phosphate, intake was increased in the first three days of life when standardized PN solutions were received, while magnesium and calcium intake were marginally reduced at day 1 of life (Figure 2a–f, Supplementary Table S4).

### 3.2. Balanced Indicators of Metabolism with Standardized PN Solution

Despite the increased intake of carbohydrates, blood glucose concentrations did not differ (Figure 1i) significantly between the study and the reference cohort. Likewise, pH levels did not differ significantly between the two cohorts. As a critical readout for bone metabolism, calcium and phosphate supply and excretion levels were monitored and demonstrated a reduced variability of the relative calcium to phosphate intake in the standardized PN solution cohort (study cohort (*MUC PREPARE*): 1.30 ± 0.17 vs. reference cohort: 1.89 ± 0.67; mean ± SD; Leven test *p*-value: 5.81 × 10^−6^). Serum sodium levels were decreased in the first three days of life in the study cohort (Figure 2g), while potassium (Figure 2h), and phosphate (not shown) were comparable between the study and the reference cohort.

**Figure 3:**
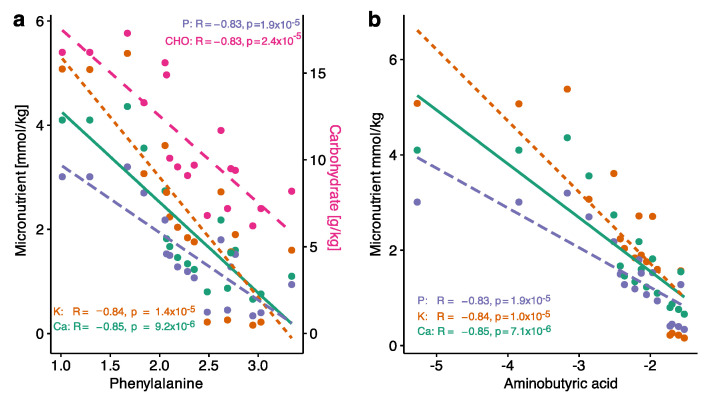
Scatterplots of Pearson correlations of (**a**) phenylalanine with calcium, potassium, phosphate, and carbohydrate (g/kg) intake levels and (**b**) of ABA with calcium, potassium, and phosphate levels. All *p*-values were significant after FDR.

Metabolomic profiles in the study cohort were not altered by the prevalence of infection, both early and late onset, RDS, BPD, or nutritional intake, i.e., total volume (mL), calories, macronutrient supply, i.e., lipid, protein, and carbohydrate supply all expressed as either g/kg or % of total calorie intake, and micronutrient supply, i.e., natrium, magnesium, and chloride supply. We did show, however, significant inverse correlations of phenylalanine and alpha-aminobutyric acid levels with calcium, potassium, and phosphate intake after correcting for multiple testing using FDR. In addition, phenylalanine was inversely correlated with total carbohydrate intake (g/kg) (Figure 3). There was no correlation of phenylalanine to protein intake and total volume; negative associations with lipid intake (β = 2.19, *p* = 0.00088, FDR adjusted *p* = 0.23) and calorie intake (β = −39.28, *p* = 0.0005, FDR adjusted *p* = 0.13) did not remain significant after multiple testing.

### 3.3. Improved Rate of Enteral Feeding and Postnatal Growth with Standardized PN Solutions

**Figure 4:**
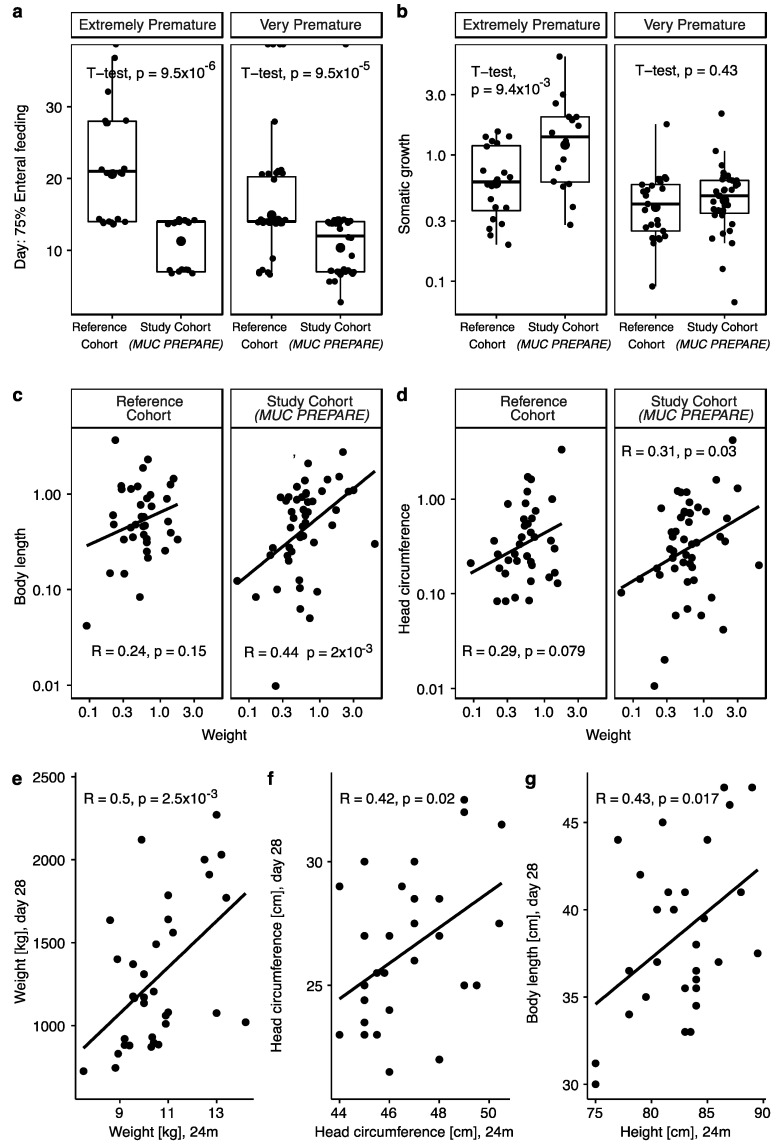
(**a**) Days of life by when infants achieved 75% enteral feeding in percentage of total volume (mL) given separated by immaturity. (**b**) Somatic growth calculated as change in percentiles at day of birth and day 28 of life separated by immaturity. (**c**,**d**) Scatterplots showing Pearson correlations of weight with (**c**) body length and (**d**) head circumference separated by cohort. (**e**–**g**) Scatterplots showing Pearson correlations over both cohorts in available data showing that the (**e**) body weight (kg), (**f**) head circumference (cm), and (**g**) height/body length (cm) correlate with the respective values at 24 months. Extremely immature: <27 GA weeks, very premature: 27–32 GA weeks. Study cohort (*MUC PREPARE*): 2012–2014, reference cohort: 2007–2009.

All infants in the standardized PN cohort achieved earlier enteral feeding (75% of total fluid intake; study cohort (*MUC PREPARE*): 10.6 ± 3.6 days; reference cohort: 17.2 ± 6.6 days; mean ± SD, *p* = 1 × 10^−8^, Supplementary Figure S1A) and earlier termination of any PN (study cohort: 17.4 ± 11.7 days; reference cohort: 22.4 ± 12.7 days (mean ± SD), *p* = 0.038, Supplementary Figure S1B). Infants receiving the *MUC PREPARE* achieved 75% enteral feeding on average 4 days (27–32 weeks GA) and 1.5 weeks (<27 weeks GA) earlier when compared to the infants that received individual PN (<27 weeks GA: study cohort: 11.3 ± 3.5 days; reference cohort: 20.6 ± 7.1 days; *p* = 9.5 × 10^−6^, 27–32 weeks GA: study cohort: 10.4 ± 3.6 days; reference cohort: 14.9 ± 5.1 days; *p* = 9.5 × 10^−5^; all values mean and SD; Figure 4a).

Regarding somatic growth, infants that received standardized PN solutions (*MUC PREPARE*) showed significantly improved weight gain when compared to the reference cohort, with most significant effects in the most premature infants (study cohort: 1.63 ± 1.42; reference cohort: 0.72 ± 0.45 (difference in percentiles at day of birth and day 3 of life; mean ± SD), *p* = 0.0094; Figure 4b). There was no significant difference in initial weight loss between the two cohorts independent of immaturity at birth. Feature selection showed that—regardless of cohort—the main factor influencing weight gain and body length growth was gestational age, while prematurity was a factor for body length where the younger infants grew more (Supplementary Table S5).

In the study cohort with standardized PN (*MUC PREPARE*), weight gain was correlated with an increase in body length (R = 0.44, *p* = 0.002, Figure 4c) and head circumference (R = 0.31, *p* = 0.03; Figure 4d), in contrast to the lack of correlation in the reference cohort.

Growth parameters at 28 days of life in the study cohort correlated significantly with the respective growth parameter at 24 months corrected age (weight (R = 0.5, *p* = 0.0025), body length/height (R = 0.43, *p* = 0.017), head circumference (R = 0.42, *p* = 0.02)) (Figure 4e–g). Supplementary Table S5 summarizes the results.

## 4. Discussion

The aim of standardizing PN is to increase patient safety and to optimize resource efficiency by minimizing procedural incidents when providing PN to meet recommendations and individual patient needs [31]. To address these vital issues, guidelines, and recommendations for PN of preterm infants have continued to change and evolve over the past decades, requiring perinatal centers to adapt their infusion regimens accordingly [18,20,21,22,32] while meeting increasingly higher hygiene standards.

In a representative cohort of preterm infants, we demonstrated that our standardized PN solutions for preterm infants *MUC PREPARE* resulted in an improved energy and macro- as well as micronutrient intake meeting international recommendations [24]. Optimized PN resulted in a balanced metabolic profile, earlier enteral nutrition, and improved somatic growth.

While commercially available PN solutions for infants generally cover the needs of a broader range of age groups and diseases and are not adapted to the different postnatal growth phases, we have developed standardized PN solutions especially for the growing, high-risk cohort of patients after premature birth (Munich Preterm Parenteral feeding solution (*MUC PREPARE*)). The ‘one-fits-all’ policy of commercial products often requires either an increase in fluid intake or a restriction of energy or micronutrient intake when conditions are critical after preterm birth. Most of these commercial solutions are designed for administration via central infusion lines [33] or require individual dilution of the solution, thereby risking calculation errors and/or non-optimal macro- and micronutrient intake. The need for adding vitamins and micronutrients to the commercial solutions also increases the likelihood of calculation errors and hygienic risks [34]. The *MUC PREPARE* solutions enable standardized, but individually adaptable nutrition in the first weeks of life through nine different formulations and meet the highest quality criteria regarding nutrient content and application safety. In contrast to commercially available nutrition solutions, our PN solutions are adapted to the higher macro- and micronutrient requirements with the simultaneous restriction of fluid intake, characteristic for infants with significant immaturity.

### 4.1. Improved Early Postnatal Energy and Nutrient Supply with the Standardized PN Solution MUC PREPARE in Very Preterm Infants

Postnatal nutrient intake is crucial for somatic growth [35]. Improved nutrient intake not only leads to a faster regain of birth weight but was shown to improve somatic growth including body weight and head circumference until term in infants with a birth weight of less than 1500 g [36]. Even small differences in the supply of macro- and micronutrients were shown to have significant effects on growth and metabolism [37,38] best exemplified by studies demonstrating that protein intake the first week of life determines insulin sensitivity through epigenetic changes [39] and significantly affects cognitive performance at 18 months of age [40].

Without increasing overall fluid intake, *MUC PREPARE* was able to improve nutritional intake according to the ESPGHAN recommendations [24] when compared to the group of infants receiving individually prescribed PN. This included a higher calorie intake and a balanced increase in macronutrient supply leading to significantly increased carbohydrate, protein, and lipid intakes (g/kg) as early as day three of life. Here, especially improved amino acid intake critically impacts on somatic and organ growth but requires improved levels of overall macronutrient intake to balance energy consumption and to ensure adequate growth in preterm infants at the same time. This is in line with previous publications favoring a standardized PN regimen [34,37,41,42]. In contrast to these studies, however, *MUC PREPARE* resulted in a higher calorie intake [43] and improved growth shown across a large set of study time points [41,42] and even meets updated ESPGHAN recommendations [18]. In addition, micronutrient intake was increased, and serum levels showed less fluctuations under *MUC PREPARE* PN in comparison to the reference cohort. Stabilization of micronutrient intake as achieved when receiving *MUC PREPARE* is important when considering publications demonstrating deranged electrolyte levels and later disease development, specifically when considering sodium intake in preterm infants and the later development of arterial hypertension [44]. When considering critical goals for bone growth and mineral density in preterm infants [45,46,47,48], calcium and phosphate intake and excretion were stabilized and more likely within the recommended calcium/phosphate ratio of 1.3–1.7 [43] when receiving *MUC PREPARE*, in line with recommendations from ESPGHAN and the American Academy of Pediatrics, despite the actual supply being still lower than currently recommended.

### 4.2. Balanced Metabolomic Profile with the Use of a Standardized PN Solution

Despite increased macronutrient intake, blood glucose concentrations and pH levels did not differ significantly between the study and reference cohorts, indicating a balanced metabolism, reflecting previous studies [49,50]. The metabolic profile, including amino acids, biogenic amines, lipids, and acylcarnitines, was unaffected by the remaining variability of nutrient intake within *MUC PREPARE* study infants. In contrast to other studies, postnatal complications or degree of immaturity did not alter the metabolomic profiles [51]. The only exception were differentially co-regulated levels of the amino acids alpha-aminobutyric acid and phenylalanine with macro- and micronutrient intake, in line with previous studies indicating negative correlations with energy supplementation [52] but indicating lower risk profiles for morbidity and mortality [53,54,55] in infants with optimized PN at the same time.

### 4.3. Improved Rate of Enteral Nutrition and Postnatal Growth with Standardized PN Solutions

Referring to the two most important outcome measures, we demonstrated a reduced time until full enteral feeding as well as improved weight gain in infants receiving optimized PN with *MUC PREPARE.* Early enteral feeding is known to reduce complications caused by parenteral infusion therapy [21] and to increase postnatal energy intake and weight gain [56]. The beneficial effects of adequate weight gain related to expected growth in utero include lower rates of complications of necrotizing enterocolitis (NEC), bronchopulmonary dysplasia (BPD), and nosocomial sepsis [3,4], as well as improved neurological outcome until preschool age [8,57,58] and a reduced rate of metabolic diseases in adulthood [59,60]. Notably, the beneficial effects on weight gain were most pronounced in the most immature infants and, in addition, were long lasting when growth parameters were followed up to 2 years of age. The correlations of body length and head circumference to weight gain reflects balanced somatic growth and underlines the lasting effects of nutrition in this group.

The limited number of patients studied is due to the character of the cohort including only very preterm infants. The limitation was counteracted by the homogeneity of each cohort due to pairwise matching as shown by the significant comparability for perinatal complications between the study and the reference cohort. Despite possible differences in postnatal care in general and changes in mechanical ventilation duration in the study cohort, this difference must be considered when interpreting study results (study cohort (*MUC PREPARE*), reference cohort). A second limitation is the changes in the number of parenterally fed infants over time, with an increasing number of infants achieving full enteral feeding as expected, particularly in the study cohort. This must be considered when interpreting the results from day 7 onwards, but at the same time reflects a significant advantage of the standardized PN regimen.

## 5. Conclusions

In summary, the standardized infusion regimen *MUC PREPARE* appears to be safe and beneficial for meeting nutrient needs and supporting growth, when compared to individually prescribed and compounded PN. We demonstrated a significant increase in energy and macro- and micronutrient supply together with a balanced metabolic profile, earlier achievement of full enteral nutrition, and improved short- and long-term growth. The standardized infusion concept is thus recommended for clinical use as a superior PN strategy in very preterm infants [61]. Broad application of standardized PN concepts may also facilitate comparability between perinatal centers and thereby improve outcome assessment in multicentric clinical trials.

## Figures and Tables

**Table 1:** Patient characteristics.

	Study Cohort (*MUC PREPARE*)			Reference Cohort		
	All (*n =* 58)	<27 Weeks (*n =* 18)	> 27 Weeks (*n =* 40)	All (*n =* 58)	<27 Weeks (*n =* 22)	>27 Weeks (*n =* 36)
Gestational age [weeks PMA]	27.8 ± 2.31	25.1 ± 1.03	29.1 ± 1.40	27.8 ± 2.23	25.5 ± 1.14	29.3 ± 1.23
Birth weight [gram]	989 ± 415	647 ± 415	1144 ± 655	991 ± 535	727 ± 535	1152 ± 550
Maximal weight loss [% to birth weight]	−1.18	−1.37	−5.32 ± 4.17	−0.66	−0.64	−0.64
Gender [female, male]	34/24	10/8	24/16	34/24	12/10	22/14
BPD (none/all grades) *	33/23	7/11	28/12	49/7	17/5	34/2
RDS ≥ grade 3	8 (13.79%)	3 (16.67%)	5 (12.5%)	8 (13.79%)	7 (31.82%)	1 (2.78%)
IVH ≥ grade 3	2 (3.45%)	0 (0%)	2 (5%)	0 (0%)	0 (0%)	0 (0%)
PDA	21 (36.21%)	12 (66.67%)	9 (22.5%)	30 (51.72%)	20 (90.91%)	10 (27.78%)
NEC	1 (1.72%)	1 (5.56%)	0 (0%)	1 (1.72%)	1 (4.55%)	0 (0%)
Early onset infection	11 (18.97%)	6 (33.33%)	5 (12.5%)	6 (10.34%)	3 (13.64%)	3 (8.33%)
Late onset neonatal sepsis	16 (27.59%)	9 (50%)	7 (17.5%)	18 (31.03%)	11 (50%)	7 (19.44%)
Invasive ventilation [days]	20.7 ± 13.7	28 ± 14.1 *	18.1 ± 12.7	22.1 ± 19.1	41.5 ± 14.6 *	12.8 ± 13.1
Non-invasive ventilation [days]	10.5 ± 12.4	17.2 ± 15.0	5.58 ± 7.13	12.5 ± 11.4	17.2 ± 12.2	6.5 ± 6.43
Oxygen supplementation [days]	30.1 ± 26.9	43.9 ± 31.1	21.8 ± 20.6	34.3 ± 35.1	58.7 ± 36.7	14.4 ± 17.2

Data are given as mean ± standard deviation or percent of total in group [*n* (%)]. PMA: postmenstrual age, RDS: respiratory distress syndrome, IVH: intraventricular hemorrhage, PDA: patent ductus arteriosus, BPD: bronchopulmonary dysplasia. * indicates significant differences between the two cohorts.

**Table 2:** ‘NeoPeri’ and ‘NeoZent’ solutions containing amino acids, glucose, electrolytes, and trace elements, supply per kg bodyweight per day.

	Baxter AminopädR 10%	Dextrose	Potassium	Sodium	Calcium	Phosphate	Magnesium	Zinc Total	Baxter Addel Junior	Chloride	Volume	KJ	Osmolarity
Per Day/kg BW	g	g	mmol	mmol	mmol	mmol	mmol	µmol	mL	mmol	mL	KJ	mOsm
NeoPeri-1	2.3	7	0	1	1	0.5	0	0	0	0	80	158	731
NeoPeri-2	2.5	8	1	2.6	1.3	1.3	0.5	7.3	1	1	97	179	735
NeoPeri-3	3.5	8.5	1.5	3	1.5	1.5	0.5	7.3	1	1.5	115	204	727
NeoZent-1 G(−)	3	6	0	1	1	0.5	0	0	0	0	46	153	1231
NeoZent-1 G(±)	2.5	6.5	0	1	1	0.5	0	0	0	0	47	153	1238
NeoZent-2 G(−)	3.5	7	1	2.6	1.5	1.3	0.5	7.3	1	1	62	179	1205
NeoZent-2 G(±)	3	8	1	2.6	1.5	1.3	0.5	7.3	1	1	61	187	1226
NeoZent-3 G(−)	3.8	6.5	1.5	3	1.5	1.5	0.5	7.3	1	1.5	66	175	1179
NeoZent-3 G(±)	3.8	8.5	1.5	3	1.5	1.5	0.5	7.3	1	1.5	70	209	1229

**Table 3:** ‘NeoPeri’ and ‘NeoZent’ solutions containing amino acids, glucose, electrolytes, and trace elements, supply per 100 mL solution.

Per 100 mL	Baxter AminopädR 10%	Dextrose	KCl 7.45%	NaCl 5.85%	Calcium Solution 10%	Glycero-PO_4_-Na 1 M	Mg 0.3 M	Unizink	Baxter Addel Junior	Water for Injection
	mL	mL	mL	mL	mL	mL	mL	mL	mL	mL
NeoPeri-1	28.8	17.53	0	0	5.45	0.63	0	0	0	47.59
NeoPeri-2	25.73	16.47	1.03	0	5.82	1.34	1.75	0.51	1.03	46.32
NeoPeri-3	30.51	14.82	1.31	0	5.68	1.31	1.48	0.44	0.87	43.58
NeoZent-1 G(−)	64.04	25.62	0	0	9.29	1.06	0	0	0	0
NeoZent-1 G(±)	53.94	28.05	0	0	9.39	1.07	0	0	0	7.55
NeoZent-2 G(−)	56.43	22.57	1.61	0	10.51	2.1	2.74	0.81	1.61	1.61
NeoZent-2 G(±)	48.76	26.01	1.63	0	10.6	2.11	2.76	0.81	1.63	5.69
NeoZent-3 G(−)	57.38	19.63	2.27	0	9.85	2.27	2.57	0.76	1.51	3.78
NeoZent-3 G(±)	54.5	24.38	2.15	0	9.35	2.15	2.44	0.72	1.43	2.87

**Table 4:** ‘NeoFett’ solutions, containing fat and vitamins, supply per kg bodyweight per day.

Per Day/kg BW	Fat 20% ^1^	Baxter SoluvitR N (In Vitalipid)	Baxter Vitalipid Infant	Vol	KJ
	g	mL	mL	mL	KJ
NeoFett Low 1 ± Vit	1	1	3	6.5	42
NeoFett Med 2 ± Vit	2	1	3	11.5	84
NeoFett High 3 ± Vit	3	1	3	16.5	125

^1^ Clinoleic or SMOF-Lipid.

**Table 5:** ‘NeoFett’ solutions, containing fat and vitamins, supply per 100 mL.

Per 100 mL	Fat 20% ^1^	Baxter SoluvitR N (In Vitalipid)	Baxter Vitalipid Infant
	mL	mL	mL
NeoFett Low 1 ± Vit	53.85	15.38	46.15
NeoFett Med 2 ± Vit	73.91	8.7	26.09
NeoFett High 3 ± Vit	81.82	6.06	18.18

^1^ Clinoleic or SMOF-Lipid.

## Data Availability

The data presented in this study are available on request from the corresponding author. The data are not publicly available due to privacy reasons.

## Supplementary Materials

The following supporting information can be downloaded at: https://www.mdpi.com/article/10.3390/nu14193912/s1, Table S1: Overview of recommendations per reference cohort and feeding mode; Table S2: Summary table of provided nutrients; Overview of all macro- and micronutrients measurements available over all timepoints and infants that are below, within, and above the excretion guidelines per age group and birth cohort. The recommendations are outlined against the birth cohorts and values investigated, as they increased over the years; Table S3: Overview of fold changes, means, SD and n for each variable shown in Figure 1; Table S4: Overview of fold changes, means, SD and n for each variable shown in Figure 2; Table S5: Overview of feature selection. Figure S1A: Day of 75% enteral feeding, Figure S1B: Day of 100% enteral feeding.
