# Supplementary figures and images for: Improved Macro- and Micronutrient Supply for Favorable Growth and Metabolomic Profile with Standardized Parenteral Nutrition Solutions for Very Preterm Infants
Source: Nutrients. 2022 Sep 21;14(19):3912. doi: 10.3390/nu14193912 (PMC9572167; doi:10.3390/nu14193912)

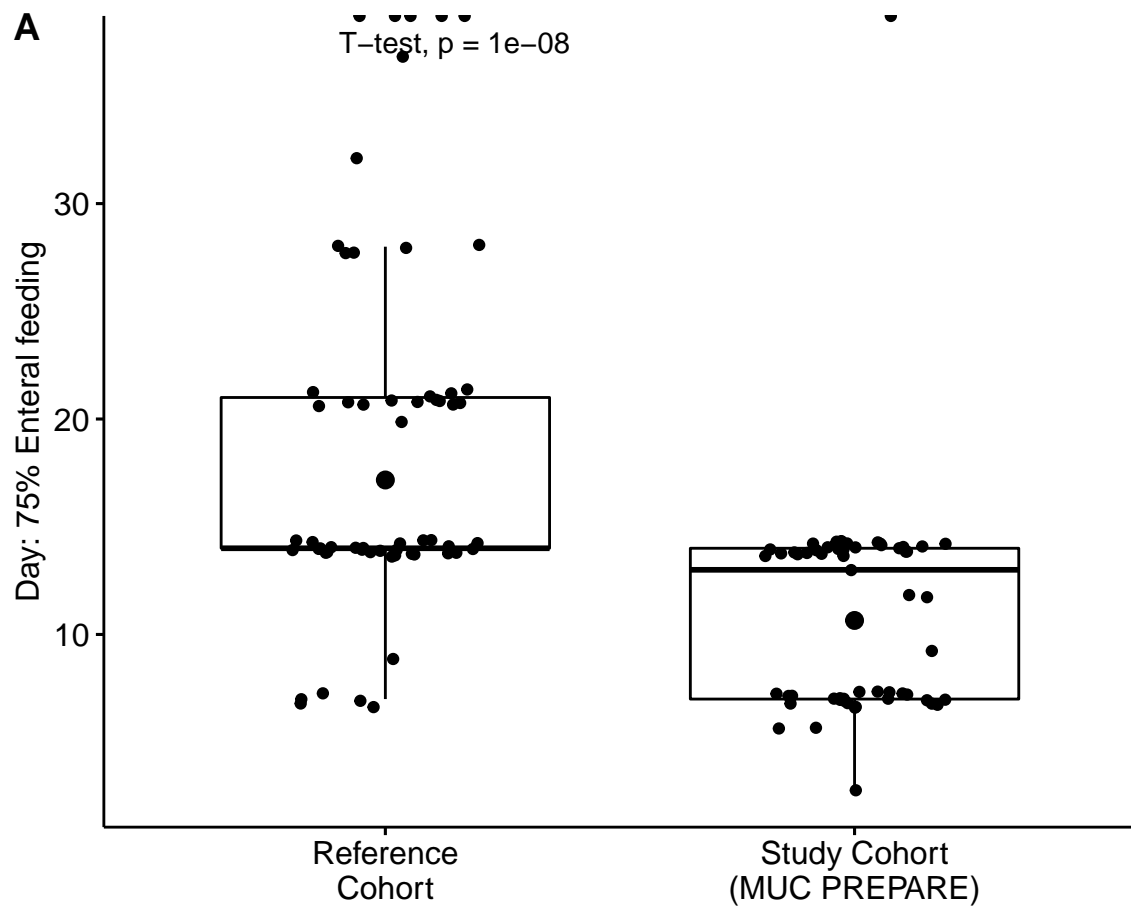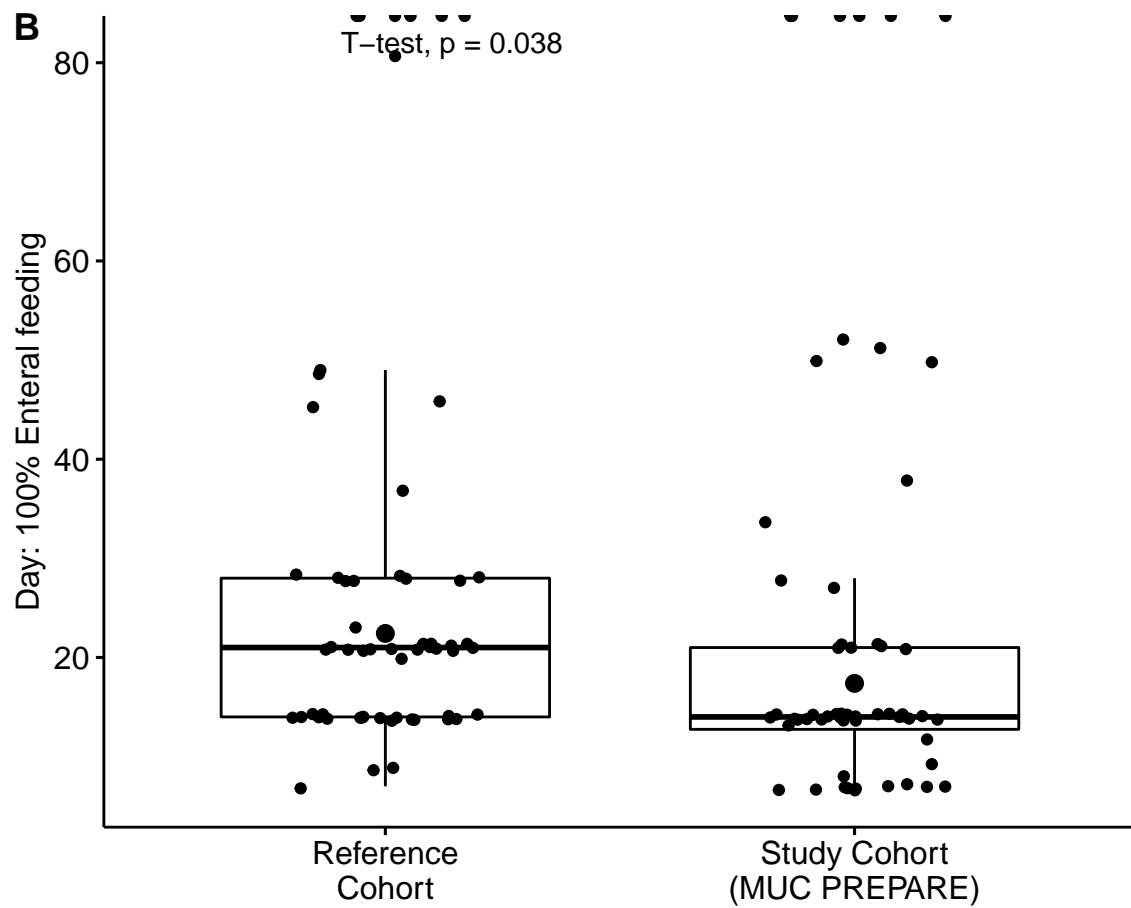

Supplement: Supplementary file 1 [file nutrients-14-03912-s001.zip › Supplemental_Figure S1.pdf]
